# Interpersonal Dynamics in 2-vs-1 Contexts of Football: The Effects of Field Location and Player Roles

**DOI:** 10.3389/fpsyg.2019.01407

**Published:** 2019-07-03

**Authors:** Timo Laakso, Keith Davids, Jarmo Liukkonen, Bruno Travassos

**Affiliations:** ^1^ Department of Sport Sciences, University of Jyväskylä, Jyväskylä, Finland; ^2^ Centre for Sports Engineering Research, Sheffield Hallam University, Sheffield, United Kingdom; ^3^ CreativeLab, Research Center for Sports Sciences, Health Sciences and Human Development (CIDESD), Universidade da Beira Interior, Covilhã, Portugal

**Keywords:** football, patterns of play, affordances, effectiveness, players’ roles

## Abstract

This study analyzed the spatial-temporal interactions that sustained 2-vs-1 contexts in football at different field locations near the goal. Fifteen male players (under 15 years, age 13.2 ± 1.03 years, years of practice 4.2 ± 1.10 years), 5 defenders, 7 midfielders, and 3 attackers, participated in the study. Each participant performed a game to simulate a 2-vs-1 sub-phase as a ball carrier, second attacker, and defender at three different field locations, resulting in a total number of 142 trials. The movements of participants in each trial were recorded and digitized with TACTO software. Values of interpersonal distance between the ball carrier and defender and interpersonal angles between players and between the goal target, defender, and ball carrier were calculated. The results revealed a general main effect of field location. Generally, the middle zone revealed the lowest values of interpersonal distance and angle between players and the right zone and the highest values of interpersonal distance between players and interpersonal angle between players and the goal. Related with participants’ roles, defenders revealed subtle differences as attackers on interpersonal distances and relative angles compared with midfielders and attackers. Findings supported that field location is a key constraint of players’ performance and that players’ role constraint performance effectiveness in football.

## Introduction

Team sports have been investigated, as complex adaptive systems, with the aim of describing and explaining emergent behaviors of players from an ecological dynamics perspective. This approach requires analysis of the continuous interactions between attacking and defending players who, fundamentally, compete to gain/retain possession of the ball and move it into favorable attacking positions in critical scoring spaces in the playing area (Araújo and Davids, 2016). McGarry (2009) highlighted the dynamical nature of these continuous interactions, which can be observed at different levels of analysis from the entire competitive context to relevant game sub-phases (i.e., 1-vs-1, 2-vs-1, 3-vs-2, etc.). For this reason, a team game has been conceptualized as a complex adaptive system whose behaviors are driven or perturbed by interactions of multiple, smaller sub-systems composed of attackers and defenders interacting under constraints (Travassos et al., 2013b). For instance, research has highlighted specific contextual performance constraints that change the emergent behaviors of players and teams. These task constraints include the number of players involved (Silva et al., 2014), the field dimensions (Vilar et al., 2014a), the number of goals (Travassos et al., 2014a), or even contextual performance constraints such as game pace or match outcome (Sampaio et al., 2013).

In line with the ecological dynamics perspective, the adaptive behaviors of players and teams to constant changes in contextual constraints is a result of information exchanges among the competing and cooperating players in relation to game demands (Travassos et al., 2012; Folgado et al., 2018). That is, players and teams constantly interact to create information, make decisions, and organize actions when functioning as a team during competitive performance. This view of competitive performance in teams, in ecological dynamics, is based on the sharing of spatial-temporal information that continuously supports the utilization of individual, sub-group, and team affordances (i.e., possibilities or opportunities for action to achieve a specific performance goal) (Silva et al., 2013). For each individual, as well as collective sub-systems, evidence has revealed that affordances are sustained by variations in space-time relations defined by the co-positioning of teammates and opponents, co-variations in their displacement trajectories and their movement velocities with respect to field markings and dimensions, and the location of scoring targets like goals, baskets, and try lines, for example (Vilar et al., 2012b; Silva et al., 2013; Gesbert et al., 2017). Also, players who have different team roles usually exhibit different physical, technical, and tactical capabilities (also effectivities) during performance (Varley et al., 2017; Lovell et al., 2018) and, consequently, explore and use the space-time relations in a different way for the identification of affordances for play (Laakso et al., 2017; Baptista et al., 2018). Previous research revealed that manipulating players’ roles constraint the spatial-temporal patterns of play from 1-vs-1 (Laakso et al., 2017) to 7-vs-7 (Baptista et al., 2018).

Research investigations have explored and exemplified these ideas in many different team sports including basketball (Araújo et al., 2006; Esteves et al., 2012), rugby union (Passos et al., 2008), Futsal (Travassos et al., 2012; Vilar et al., 2013b), and also in association football (Duarte et al., 2012; Clemente et al., 2013; Laakso et al., 2017).

In the context of association football, research findings have revealed that attackers need to lead the interactions in spatial-temporal relations with defenders, by promoting unpredictable changes in the values of key variables such interpersonal distance, relative angles with players and with the goal, and relative velocity to achieve successful outcomes (Schulze et al., 2018). On the other hand, defenders try to constrain attackers’ actions and maintain spatial-temporal equilibrium with them to enhance sub-system stability and successfully perform (Duarte et al., 2012; Clemente et al., 2013). That is, evidence suggests how attackers vary key movement displacement parameters to de-stabilize an “unwanted” symmetrical relationship with a marking defender in a dyad. In contrast, defenders use actions to maintain system stability and prevent attackers from breaking up their temporary dyad.

As previously reported, the field location of these ongoing interactions has a substantial effect to constrain the spatial-temporal relations in attacker-defender dyadic systems (Headrick et al., 2011; Vilar et al., 2012c; Laakso et al., 2017). Variations in proximity to the goal area or in field “longitudinal corridors of play” (middle or wing zones) result in emergence of different coordination dynamics of key variables like relative distance and the angle between an attacker and defender in relation to the goal (Headrick et al., 2011; Laakso et al., 2017). Although the effects of these constraints are clear, previous studies have mainly reported their influence in 1-vs-1 sub-phases of play.

In most team games, attackers try to gain an advantage by rapidly creating a temporary numerical overload against defenders in a specific location of the field. Particularly in association football, the creation of offensive or defensive numerical superiority near the ball is directly related to successful performance in terms of attacking space behind a defensive line or in recovering the ball (Vilar et al., 2013). Thus, the 2-vs-1 sub-phase is the minimum sub-phase of game that represents such numerical (overload) advantage to an attacking team. During this sub-phase, the ball carrier and the support attacker need to manage the spatial-temporal relations with an immediate opponent to support emergence of two possibilities for action: (s)he can dribble and face the defender in a 1-vs-1 if the defender is protecting a passing line to the second attacker or (s)he can draw the defender and pass the ball to the support attacker if a passing line emerges by the defender being drawn toward the ball dribbler. Despite its relevance for understanding the spatial-temporal changes that support the emergence of possibilities for action in overloads, little research has been conducted to observe actual competitive interactions during performance in this important sub-phase. In addition, there is a need to improve understanding of how interpersonal patterns of coordination between attackers and a defender in 2-vs-1 sub-phases are influenced by field location effects relative to the goal. A key issue is whether a defender changes co-positioning behavior, when constrained by the field location in football. Clear implications for practice could result from this study. The implications of the manipulation of the relative position of the goal target (Coutinho et al., 2018) in relation to the 2-vs-1 sub-phases or the attacker-defender participants’ performance roles (Laakso et al., 2017) allow coaches to improve the design of practice tasks according to the planned goals. Also, in line with previous studies, this study will allow to identify the task constraints that coaches can stress to improve players’ decision and action according to each task condition (Correia et al., 2012). Thus, the aim of this study was to analyze the adaptive behaviors of players who sustained 2-vs-1 sub-phases in football at different field locations near the goal (left, middle, and right zones on field) and manipulate participants’ team performance roles (i.e., divided into roles as attackers, midfielders, and defenders). In line with previous research in 1-vs-1 sub-phases (Laakso et al., 2017), we expected to observe changes in interpersonal distances and relative angles between players and the goal at different field location with high correlations between interpersonal distances and angles for right and left zones and low correlations in middle zone. Also, it was expected changes in interpersonal distances according to participants’ team performance roles as attackers or defenders on the emergent spatial-temporal patterns of interaction in the 2-vs-1 sub-phase.

## Materials And Methods

### Participants

Fifteen male players (under 15 years, age 13.2 ± 1.03 years, years of practice 4.2 ± 1.10 years) participated in this study. The sample size was calculated with G*Power (Version 3.1.5.1 Institut für Experimentelle Psychologie, Düsseldorf, Germany) for an effect size of 0.7, an α of 0.05, and a power of 0.8 (1–β). The total sample size computed by this method was a minimum of 15 players with a statistical power of 82.4%.

For the purposes of analysis, with the advice of the coaching staff, the participants were categorized into their main team performance role, resulting in sub-samples of five defenders (center-backs and full-backs), seven midfielders (center midfielders, lateral midfielders), and three attackers (forwards). All players belong to one youth team competing in a national Finnish level (2016/2017 season). All participants were right-footed and played in the first team of the club. The participants participated in five training sessions per week (90 min per session) and played an official competitive match at the weekend. The club, all parents, and the participants provided prior informed and written consent for participation in the study. The study was approved by the Ethics Committee of University of Jyväskylä according to the Declaration of Helsinki.

### Task and Procedures

**Figure 1:**
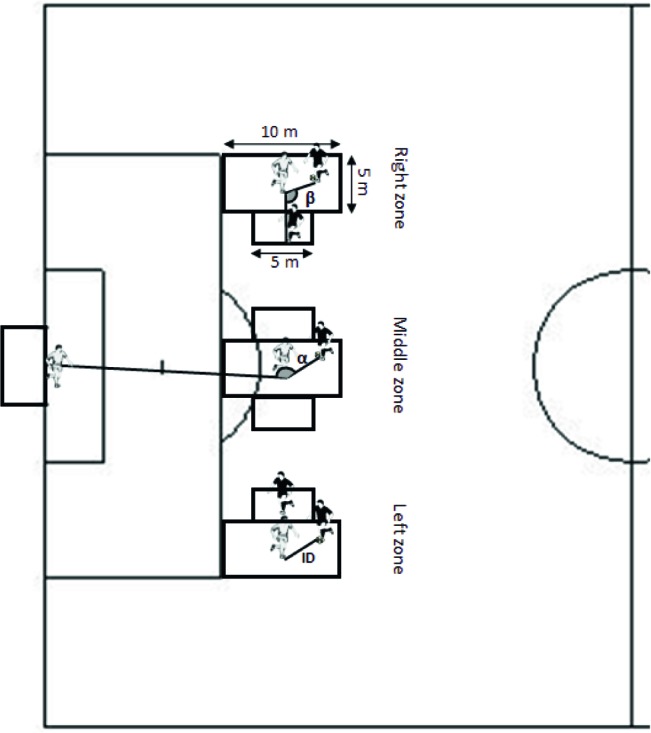
Representation of the three areas of play (left, middle, right) of ball carrier and defender and the area of second attacker and their location in relation to the goal. α – interpersonal angle between ball carrier, defender and second attacker (IABDA); β – interpersonal angle between goal target, defender and ball carrier (IAGDB); ID – interpersonal distance between ball carrier and defender.

All players were tested during four sessions in 1 week of summer break of competitive season (July) in an artificial grass pitch. The temperature was about 17–19°. The first session was used for the players being familiarized with task conditions in all field zones, and the three next sessions were used for testing purposes. Each participant performed in a game to simulate a 2-vs-1 sub-phase as a ball carrier, second attacker, and defender at three different field locations. The 2-vs-1 sub-phases occurred in a predefined area of 10 × 5 m (Passos et al., 2008; Headrick et al., 2011) in three different field locations (Left, middle and right) under competitive performance conditions (See Figure 1). The task constraints included a regular size goal (2.44 m × 7.32 m) with a goalkeeper. The starting distance between attacker and defender was 3 m. When performing in the left or on the right side of the field, the second attacker was placed in the inner side of the field in order to maintain free the wing for a possible dribble. That is, when in the right-side zone of the field, the second attacker was placed to the left of ball carrier, and when in the left zone, to the right of ball carrier. In the middle zone, the second attacker was placed at the side of the first attacker’s non-dominant foot. The area for the second attacker to move was 5 × 1.30 m (Figure 1). Before practice, all the players were informed about the rules and the goals of the tasks and encouraged to compete like in the game. The goalkeepers were also informed to act as in a competitive game. No coach feedback or encouragement was allowed during the conditions.

Before data collection, all participants engaged in a thorough warm-up routine. Each trial started when both the attacking and defending participants were ready in their starting positions and the attacking participant was requested to start the trial. As soon as the attacker moved the ball, the defender could start defending. After crossing the midline of the playing area (5 m from the end of attacking area), the attacker could dribble or pass the ball to the second attacker. The performance aim of attacking participants was to dribble past the defender and shoot to the goal or pass the ball to the second attacker who could shoot at goal. If these events occurred, the trial was over. The aim of the defender was to prevent the attackers from scoring a goal, within the laws of the game. The trial was considered over when defending participants intercepted the ball or when the ball moved outside the borders of the playing area. A regulation ball size 5 was used in all trials.

All participants performed the 2-vs-1 trials in all three zones acting as an attacker and as a defender, resulting in a total number of 142 trials. In each trial, two attacking players with the same positional roles attacking one defending player with a different positional role (e.g., Defender + Defender vs Midfielder or Midfielder + Midfielder vs Attacker). After each trial, the attacking teams and the opposition change to promote variability in pairs and roles in next trials. Any player performed two consecutive trials in the same zone nor playing with the same pair, or opponent. Participants rest about 3–4 min between trials to avoid fatigue. All trials were randomly allocated between left, middle, and right zones, comprising 50 trials in the left zone, 41 in the middle, and 51 in the right performance area. The experimental protocol allowed us to analyze the effects of participants’ performance roles in attack, and the distribution of trials by role was defenders (49 trials), midfielders (45 trials), and attackers (48 trials).

Participant movements were captured by using a single digital video camera (Sony HRX-MC50E) placed 4 m above ground forming an angle of approximately 45° with the longitudinal axis of the performance area to capture participant movements during the whole experimental task. All video recordings captured the displacement trajectories of all participants without moving the camera. The movements of participants in each trial were digitized with TACTO software at 25 Hz (Fernandes and Malta, 2007; Duarte et al., 2010). The displacement trajectories of the participants and the ball were tracked using a computer mouse, by following, in every frame, a working point located between players’ feet on the ground plan. After calibration of the pitch, with real measures of six control points for each zone (4 corners of the zone of play, and the two goalposts position), the *x* and *y* virtual coordinates of the players were extracted. The obtained virtual coordinates were transformed into real coordinates using the direct linear transformation method (2D-DLT) to avoid parallax error and filtered with a Butterworth low pass filter (6 Hz) to reduce the noise of the process of digitizing (Winter, 2005).

### Reliability of the Digitizing Procedure

Fifteen trials were selected at random and the displacement trajectories of attackers and defenders (*n* = 45) were re-digitized after 1 month by the same experimenter. Intra-digitizer reliability values were assessed using technical error of measurement (TEM) and coefficient of reliability (*R*) statistics (for details see Goto and Mascie-Taylor, 2007). The intra-TEM yielded values of 0.235 m (2.25%) with a corresponding coefficient of reliability (*R* = 0.991).

### Variables

According to our purposes, the interpersonal distance between the ball carrier and defender (ID) was calculated. Also, the interpersonal angles between (1) ball carrier, defender, and second attacker (IABDA) and (2) between the goal target (the center of the goal in order to maintain the reference fixed and allow a better understanding of the relationships between players and the goal), defender, and ball carrier (IAGDB) were calculated to investigate changes in interpersonal interactions between participants in the 2-vs-1 performance contexts (See Figure 1; Vilar et al., 2014a; Laakso et al., 2017).

### Data Analysis

Descriptive statistics were reported for all performance measures recorded. Comparisons between field zones and participants’ roles were assessed using standardized mean differences with 90% confidence intervals. The smallest worthwhile differences were estimated from the standardized units multiplied by 0.2 (Hopkins et al., 2009; Cumming, 2012). Effect size statistics were reported using the following ranges: trivial (0–0.19); small (0.2–0.59); moderate (0.6–1.19); large (1.2–1.99); and very large (≥2.0). Magnitudes of clear effects were considered at the following scale: 25–75%, possibly; 75–95%, likely; 95–99%, very likely; >99%, most likely (observed effects were represented by –ive and +ive directions) (Hopkins et al., 2009). Correlation values between variables were accessed through Pearson correlation using SPSS 22.0 software (IBM SPSS Inc., Chicago, USA). Thresholds for correlation coefficients (*r*) were: 0.30, small; 0.49, moderate; 0.69, large; 0.89, very large; and 1.00, near perfect (Hopkins et al., 2009).

## Results

### Effects of Field Location

**Figure 2:**
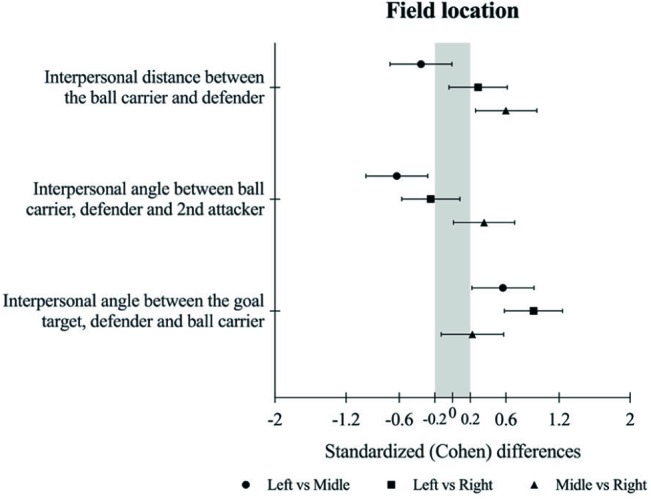
Standardized (Cohen) differences of ID, IABDA, and IAGDB for field zones (left vs. middle vs. right).

Analysis of ID revealed main effects for field zones. Small higher values were observed in comparisons of the left to middle zone (likely −ive). Moderate higher values were observed in comparisons of middle to right zone (very likely +ive). Unclear values were observed in comparisons of left to right zone (unclear). Generally, the middle zone revealed the lowest ID values, while the right zone revealed the highest ID values (see Table 1 and Figure 2).

**Table 1:** Descriptive statistics and differences in means for field location and players’ roles.

	(Mean ± SD)			Difference in means (d; 90% CL)		
Field location						
Variables	Left	Middle	Right	Left vs Middle	Left vs Right	Middle vs Right
ID (meters)	3.21 ± 1.42	2.68 ± 1.49	3.67 ± 1.73	−0.35 [−0.7–0.01]	0.28 [−0.04 0.62]	0.60 [0.26 0.95]
IABDA (degrees)	121.34 ± 20.57	107.77 ± 22.28	115.94 ± 23.22	−0.63 [−0.98–0.27]	−0.24 [−0.57 0.608]	0.36 [0.01 0.07]
IAGDB (degrees)	122.9 ± 20.76	135.72 ± 23.87	140.40 ± 17.12	0.57 [0.22 0.92]	0.91 [0.58 1.24]	0.22 [−0.13 0.57]
Players’ role						
Variables	Defenders	Midfielders	Attackers	Defenders vs Midfielders	Defenders vs Attackers	Midfielders vs Attackers
ID (meters)	3.75 ± 1.81	3.30 ± 1.59	2.77 ± 1.13	−0.26 [−0.6 0.07]	−0.65 [−0.98–0.31]	−0.38 [−0.73–0.04]
IABDA (degrees)	120.83 ± 19.88	110.45 ± 19.22	114.74 ± 26.86	−0.52 [−4.7 3.62]	−0.25 [−3.69 3.18]	0.18 [−3.29 3.66]
IAGDB (degrees)	131.71 ± 20.55	136.77 ± 20.35	130.45 ± 24.07	0.24 [−3.72 4.21]	−0.06 [−3.67 3.57]	−0.28 [−3.92 3.36]

Analysis of IABDA revealed main effects for field zones. Moderate higher values were observed in comparisons of left to middle zone (very likely −ive). Small higher values were observed in comparisons of middle zone to right zone (likely +ive). Unclear values were observed in comparisons of left to right zone (unclear). Generally, the left zone revealed higher values of IABDA, while the middle zone revealed lower values (see Table 1 and Figure 2).

Analysis of IAGDB revealed main effects for field zones. Small lower values were observed in comparisons of left to middle zone (very likely +ive). Unclear values were observed in comparisons of middle to right zone (unclear). Moderate higher values were observed in comparisons of left to right zone (most likely +ive). Generally, the right zone revealed the higher values and the left zone revealed the lower values of IAGDB (see Table 1 and Figure 2).

Analysis of relationships between ID, IABDA, and IAGDB for each field zone revealed interesting effects. In the left field zone, a large negative correlation was revealed between ID and IABDA values [*r* = −0.76, *R^2^* = 0.57 (90%CI: −0.84 to −0.64), most likely −ive], a large positive correlation between ID and IAGDB values [*r* = 0.72, *R^2^* = 0.52 (90%CI: 0.59–0.82), most likely +ive], and a moderate negative correlation between IABDA and IAGDB values [*r* = −0.46, *R^2^* = 0.21 (90%CI: −0.62 to −0.24), most likely −ive]. On the right, an unclear correlation was revealed between ID and IABDA values [*r* = 0.08, *R^2^* = 0.01 (90%CI: −0.16 to 0.31), unclear], a large negative correlation between ID and IAGDB values [*r* = −0.70, *R^2^* = 0.48 (90%CI: −0.8 to −0.55), most likely −ive], and a moderate negative correlation between IABDA and IAGDB values [*r* = −0.56, *R^2^* = 0.31 (90%CI: −0.7 to −0.37), most likely −ive]. In the middle zone, a near perfect positive correlation was revealed between ID and IABDA values [*r* = 0.93, *R^2^* = 0.87 (90%CI: 0.9 to 0.96), most likely +ive], and unclear correlations between ID and IAGDB [*r* = 0.15, *R^2^* = 0.02 (90%CI: −0.37 to 0.1), unclear] and IABDA and IAGDB values [*r* = 0.10, *R^2^* = 0.02 (90%CI: −0.14 to 0.33), unclear].

### Effects of Players’ Roles

**Figure 3:**
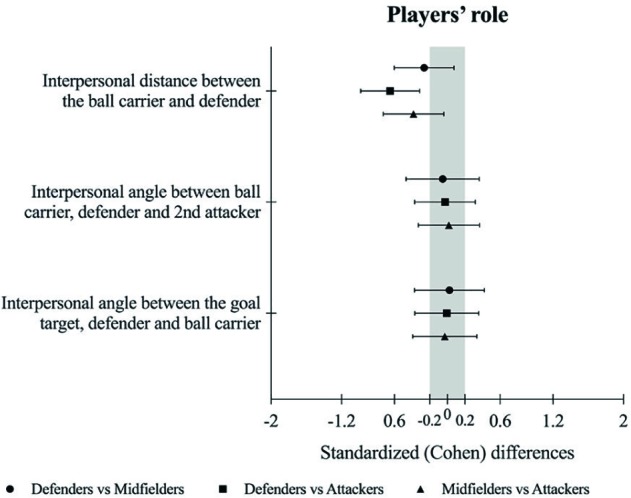
Standardized (Cohen) differences of ID, IABDA, and IAGDB for players’ role (defenders vs. midfielders vs. attackers).

Analysis of players’ roles, as attacking players, revealed subtle changes in emergent interpersonal coordination tendencies (see Table 1 and Figure 3). When defenders acted as attacking players, small higher values of ID were observed compared to midfielders (possibly −ive) and a moderate higher ID was observed compared to attackers (very likely −ive). Also, when midfielders acted as attacking players, small higher ID values were observed compared to attackers (possibly −ive). No other effects on IABDA and IAGDB were revealed in analysis of effects of players’ roles when participants acted as attacking players (see Table 1 and Figure 3). Analyses of relationships between ID, IABDA, and IAGDB for each player role were unclear.

## Discussion

The aim of the present study was to analyze the adaptive behaviors of players who sustained 2-vs-1 sub-phases in football at different field locations near the goal (left-, middle- and right- zones on field) and manipulate participants’ team performance roles (i.e., divided into roles as attackers, midfielders, and defenders). As expected, results indicated a main effect of field location and a subtle effect of participants’ roles on spatial-temporal coordination tendencies in the 2-vs-1 sub-phases. Generally, the findings reinforced effects noted in previous studies on performer interactions in football (Laakso et al., 2017).

### Effect of Field Locations

Field location was confirmed as an important constraint on interpersonal coordination of players, not just in 1-vs-1 sub-phases (Headrick et al., 2011; Laakso et al., 2017) but also in 2-vs-1 sub-phases of football. As observed in performance context (Schulze et al., 2018), according to changes in field zones of performance, the relationship between values of interpersonal distances and relative angles between players and the goal revealed different relational dynamics. Analysis of interactions in the middle zone revealed lower values for ID and IABDA (near 1–10°) and medium values (near 135°) for IAGDB. These findings contrasted with data reported in previous research on performance in 1-vs-1 sub phases, in which higher values of ID and greater relative angles between players and the goal were observed in the middle zone, compared to performance in the other zones. In 2-vs-1 sub-phases, the additional teammate increases the available affordances for the ball carrier (dribbling, shooting, passing), not allowing the defender to perform as conservatively. Thus, defenders tried to cope with the increase in affordances for attackers by dividing their efforts to occupy passing lines and inhibit the emergence of dribbling/shooting lines for the ball carrier (Vilar et al., 2012c). The near perfect correlation values observed in mid zone between ID and IABDA variables reinforced such an interpretation. Similar results have been observed in previous research (Travassos et al., 2014b; Vilar et al., 2014b).

When a defending team performing with a numerical disadvantage usually adopts a zonal defense to simultaneously occupy space and close down options for the ball carrier at the same time (Travassos et al., 2014a). That is, the defenders or even the defensive team seeks to co-position themselves to mark the opponents and the space at the same time, inhibiting the emergence of the most advantageous affordances for attackers. This strategy of defenders can be explained by an attempt to adapt to the emerging informational constraints of the 2-vs-1 sub-phase, increasing the time for ball carriers to interact, that is, explore, decide, and perform actions (van Andel et al., 2017).

Observations of performance in left and right field zones revealed contrasting findings. Analysis of performance in the left zone revealed mid values for ID, higher values for IABDA, and lower values for IAGDB. In opposition, analysis of performance in the right zone revealed higher values for ID, mid values for IABDA, and higher values for IAGDB. These performance observations may be related to the fact that all the players were right-footed, constraining possibilities for the ball carrier to explore affordances for shooting or passing, consequently allowing different affordances for defenders (Paterson et al., 2016). It is worth noting that the ball carriers’ preferred foot was the “outside” foot on the right field zone, providing the ball carrier with affordances to typically pass the defender on the right side. This affordance typically constrained the interactions for the defending players so that they could focus more on their alignment with the goal (IAGDB). These adaptations allowed defenders to maintain a large ID to provide an affordance for the ball carrier to dribble to the right and shoot at goal from the “outside” (with a narrow angle to the goal). The negative correlations between ID and IAGDB values support the use of this functional defensive strategy. It suggests that when the defender presses the ball carrier, he is seeking to maintain symmetry of the system with the goal to ensure that he could not shoot at goal with the preferred foot.

In contrast, in the left field zone, the starting position of the second attacking player was on the right side of the area. In this case, the ball carrier tried to open space to explore a dribble to the middle or to open a passing line to the second attacker. While this was happening, the defender sought to constrain the ball carrier to drive to the left and use the preferred foot and, simultaneously, seeking to occupy the passing line to the second attacker. These interactions were driven by increases in IABDA and decreases in IAGDB values. The emergent negative correlations between ID and IABDA and the positive correlations between ID and IAGDB supported the use of this defensive strategy. When a defender presses the ball carrier, a major aim is to cut the passing line from the ball carrier to the second attacker, increasing the value of IABDA and temporarily decreasing alignment with the goal. These dynamical interactions suggest that the exploration of affordances by attackers and defenders, during performance, was context-dependent and forged by variations in spatial-temporal relations between players (Vilar et al., 2012b; Silva et al., 2013; Gesbert et al., 2017) and their own effectivities (Silva et al., 2013; Paterson et al., 2016). Also, the findings clearly revealed how the location of the scoring target acted as a powerful constraint on emergent interpersonal spatial-temporal interactions of players and teams in football (Headrick et al., 2011; Vilar et al., 2012c; Laakso et al., 2017).

### Effect of Team Roles

As expected, the participants’ main performance roles constrained interpersonal coordination tendencies in the 2-vs-1 sub-phases. However, only subtle changes were revealed, particularly for defenders, compared with midfielders and attackers (Laakso et al., 2017). Compared to midfielders and attackers, defenders usually displayed different technical and tactical abilities, which constrained the identification of affordances and consequently shaped the coordination tendencies during performance (Laakso et al., 2017; Baptista et al., 2018). In this study, results revealed higher ID values for defenders, acted as attacking, compared to participants with other main performance roles. Midfielders revealed higher ID values than attackers, when acting also as attacking players. The findings suggested that the familiarity and past experience of players, acting in their main performance role or other, may influence their interaction tendencies with other participants, especially in exploiting affordances. For instance, defenders, in attack, revealed generally higher ID values than midfielders and attackers. In competition, defenders typically do not have as many opportunities to face 2-vs-1 situations near the opposite goal to achieve scoring box opportunities, as do midfielders and attackers in their team roles. Due to their typically less effective skills in attacking situations to create scoring box opportunities, defenders seek to manipulate the ball when well away from attackers. That is, defenders usually face the 2-vs-1 situations with the aim of keeping the ball possession and achieving in-depth passing opportunities, and for that, it makes sense to play with high distance from opponents to ensure secure passing lines or other options for play. In 2-vs-1 sub-phases, this lack of skill and experience may lead defenders to seek more possibilities to pass the ball to the second attacker rather than to try to dribble the defending player with the ball. These findings in contrast with previous results in a study of 1-vs-1 sub-phases show that, when defenders attack and attackers defend, lower values in interpersonal distance emerged in comparison to performance of participants with other role combinations (Laakso et al., 2017).

Our data suggest that an individual’s team role is an individual constraint that can be related to performance effectiveness (Varley et al., 2017). Due to differences in performance contexts and the requisite actions, players of different team roles exploited affordances and performed differently in competition condition (Travassos et al., 2013a; Silva et al., 2014; Araújo et al., 2017). The findings signified that participants revealed different levels of effectiveness, especially the defenders in comparison to participants with other team roles.

## Conclusions

Our findings supported the general idea that field location is a key constraint on interpersonal coordination tendencies in 2-vs-1 sub-phases of association football, as also observed in previous work on 1-vs-1 sub-phases (Headrick et al., 2011; Laakso et al., 2017). Taken together, these findings imply how coaches can design practice environments for team sport athletes. These findings in 2-vs-1 sub-phases suggested the need to analyze interactional dynamics of attackers and defenders in different relevant sub-phases of team games (i.e., 3-vs-2, 3-vs-3, 4-vs-3, 5-vs-5) (Laakso et al., 2017). These observations are important to understand how manipulated constraints in team games practice can change interpersonal coordination tendencies and how players explore such variations. The results also suggested that the manipulation of different field playing locations should be promoted in practice. Further research is also required to understand the dynamics of this game sub-phase during training sessions or in the game environment. That is, what is really the transfer between such spatial-temporal coordination tendencies in training and competition and how it happens at different levels of relations (from individuals to teams).

The manipulation of the relative position of the goal could highlight the behavior of defenders to effectively manage the spatial-temporal relations with opponents and constrain affordances according to the current effectivities (capacities) of players (for instance use of a preferred foot). Such manipulations have implications for specificity of practice, highlighting the importance of conditioning for footwork and management of spatial-temporal relations with opponents, which can be best attained in sub-phase practices (rather than ladder drills) because of the perception of information for action (affordances).

Despite the obtained results, some limitations should be acknowledged. In this study, only U15 players from one team were considered for analysis. Further research should be developed using larger sample of players and considering diferent ages and levels of practice to identify variations or similarities between spatial-temporal coordination tendencies. Also, independently of the age and level of practice, further studies should evaluate the technical/tactical proficiency of players and their level of fitness and maturation in order to understand the impact of individual characteristics on the spatial-temporal coordination tendencies developed in 2-vs-1 sub-phases of association football.

At the end, it was clear that changes in contextual game constraints such as relative position of the goal promote adaptive behaviors of players to perform. In line with that, coaches should constantly promote changes in the field location of 2-vs-1 sub-phases of game in order to promote the creation of new possibilities for action of players. Also, the definition of different couples of attackers and defenders according to different levels of effectivities seems to be a good constraint to create new spatial-temporal information and promote new possibilities for action of players according to their effectivities. Further research is required to understand the contribution of such manipulations to the learning process.

## Ethics Statement

The club and all parents of participants provided prior informed consent for participation in the study. The study was approved by the Local Ethics Committee according to the Declaration of Helsinki.

## Author Contributions

TL, KD, JL, and BT participated in study design. TL and JL participated in data collection. TL, KD and BT participated in data analysis and in the first draft manuscript. All the authors participated and approved the final version of the manuscript and agree with the order of the presentation of the authors.

### Conflict of Interest Statement

The authors declare that the research was conducted in the absence of any commercial or financial relationships that could be construed as a potential conflict of interest.
